# Sperm and testicular tissue cryopreservation and assisted reproductive technology outcomes in male cancer patients: a 15-year experience

**DOI:** 10.1007/s00432-022-04488-y

**Published:** 2022-11-23

**Authors:** Marta J. Fernández-González, Anne-Catherine Radauer-Plank, Cornelia Stelzer, Waldemar Geiger, Irena Goranova, Anja Borgmann-Staudt, Magdalena Balcerek, Ina Wilkemeyer

**Affiliations:** 1 Department of Pediatric Oncology and Hematology, Charité-Universitätsmedizin Berlin, Cooperation Member of Freie Universität Berlin, Humboldt-Universität zu Berlin, and Berlin Institute of Health, Berlin, Germany; 2 Clinic for Urology-Cryobank, Charité-Universitätsmedizin Berlin, Cooperation Member of Freie Universität Berlin, Humboldt-Universität zu Berlin, and Berlin Institute of Health, Berlin, Germany; 3 Berlin Institute of Health (BIH), Berlin, Germany

**Keywords:** Cancer, Male, Fertility preservation, Assisted reproduction technology, Adolescents

## Abstract

**Objective:**

To explore the characteristics of cancer patients who cryopreserved sperm/testicular tissue samples in the Cryobank of Charité-Universitätsmedizin Berlin between 2004 and 2019, and the ART utilization rate with associated outcomes.

**Methods:**

Retrospective data were available for 506 cancer patients, of which 46 (9.1%) had used their samples for artificial reproductive technologies (ART). Corresponding cycle information was collected from external fertility centers.

**Results:**

Our cohort included 53/506 (10.5%) patients aged < 18 years at diagnosis. While adolescents and adults mainly banked sperm, adolescents showed higher rates of testicular tissue cryopreservation before (11.8%, 6/51 vs. 6.4%, 26/406) and after treatment (16.7%, 4/24 vs. 7.8%, 13/167). At study conduction, storage had been ended for 44.8% (269/601) of samples. The majority of samples used for ART were requested within the first 3 years after cryopreservation (71.5%, 28/39, range = 0–12 years). Pregnancy rate was 51.4% (19/37 cycles), resulting in 11 singleton births, 3 twin pairs, and 4 miscarriages.

**Conclusion:**

With the new advantage of public health insurance coverage of fertility preservation (FP) in Germany, an increased utilization has already been noticed in our center, emphasizing the necessity of further knowledge for individual counseling. Adolescent cancer patients need to be addressed specifically, as these patients show especially low cryopreservation rates.

**Supplementary Information:**

The online version contains supplementary material available at 10.1007/s00432-022-04488-y.

## Introduction

A cancer diagnosis and its necessary treatment can impair spermatogenesis in male (long-term) survivors or even lead to irreversible infertility (Okada and Fujisawa 2018). Cryopreservation of sperm or testicular tissue are well-established techniques of fertility preservation (FP) in cancer patients and current guidelines recommend its use before initiation of a potentially gonadotoxic treatment (Dittrich et al. 2018; Lambertini et al. 2020). Despite these recommendations, the rate of FP utilization in male cancer patients is low (Balcerek et al. 2020; Chong et al. 2010). Treating physicians may experience a variety of barriers related to counseling of their patients on the risk of fertility impairment and on FP options, contributing to low FP utilization rates in patients (Halpern et al. 2020). The majority of cancer patients, including adolescents (Korte et al. 2020), however, desire to have (future) biological children (Schover et al. 2002). The inability to achieve a pregnancy may lead to reduced psychosocial well-being (Maroufizadeh et al. 2018). While natural conception following a cancer diagnosis and oncologic treatment is possible, difficulties in achieving a pregnancy remain common and survivors may require support from assisted reproductive technologies (ART). Live-birth rates following ART in cancer patients are—regardless of whether fresh or cryopreserved sperm cells are used—comparable to rates in infertile couples in Europe (Papler et al. 2021). Adequate patient counseling, early FP, and surveillance of fertility following cancer treatment are essential for successful fatherhood in cancer patients.

Until recently in Germany, FP services were not covered by health insurances. Only in a few cases, foundations supported individual patients and families. Following the initiative of the foundation for young adults with cancer (Stiftung für junge Erwachsene mit Krebs) and the German Society of Hematology and Cancer (DGHO), a health policy series on FP in patients who receive(d) gonadotoxic treatment was released in November 2017 (Bokemeyer et al. 2017). Political discussions in this context, supported by numerous medical societies, were accompanied by media reports, and eventually resulted in the amendment of the underlying law (Sozialgesetzbuch, SGB V) in 2019, which obliged health insurances to cover FP in patients who receive(d) gonadotoxic treatment. It took until 2021 to finalize interdisciplinary discussions, led by the joint federal committee (Gemeinsamer Bundesausschuss, GBA), to determine service providers and recipients and to finalize the respective billing numbers. Growing demand for cryopreservation among cancer patients, and issues regarding the implementation of the new legal situation require continuous joint efforts to enable all patients entitled to these services to have their costs covered as quickly as possible.

## Objective

The present study describes the characteristics of male cancer patients who used the FP service at the Cryobank, Clinic for Urology, *Charité-Universitätsmedizin* Berlin, Germany between 03/2004 and 05/2019, and of their cryopreserved samples. We additionally assessed utilization rates of these samples for ART and the respective outcomes.

## Material and methods

### Study population and data collection

Overall, 1073 sperm and testicular tissue samples from 919 men were cryopreserved at the Cryobank of the Clinic for Urology at the Charité-Universitätsmedizin Berlin, Germany between 03/2004 and 05/2019 (Supplement Fig. 1). Sample and patients medical data were traced from our hospital case notes from 01/2020 to 09/2021, and identified 506 cancer patients for whom oncologic treatment data were available. At the time of study conduction, 46 cancer patients (9.1%) had previously requested their samples for ART. We requested informed consent from these men to additionally obtain fertility cycle information from the respective fertility centers in Germany in which they had chosen to undergo ART, as we do not provide this service in-house. Our study was approved by the ethic committee of Charité-Universitätsmedizin Berlin (EA4/158/19).

### Cancer diagnosis and therapy groups

Underlying cancer diagnoses were classified as *hematological malignancies*, *brain tumors*, *testicular tumors,* and *non-testicular tumors.* Gonadotoxic-risk of previous cancer treatment was defined according to FP guidelines (Dittrich et al. 2018)*.*

### Semen and testicular tissue analyses and cryopreservation

Semen analyses were performed before cryopreservation according to the valid WHO laboratory manual of 1999 or 2010 (World Health Organization 1999, 2010) and assessed volume (ml), pH value, sperm concentration (10^6^/ml), motility (*a* + *b* + *c*%), and vitality (%). Men with a low sperm count or a sample volume below WHO reference value were advised to provide additional samples. In case of very low sperm counts/azoospermia, the ejaculate was centrifuged, and the sediment was cryopreserved if motile sperm were present or if patients insisted. Men with azoospermia were advised to cryopreserve testicular tissue. Testicular tissue samples (as big as a grain of rice) were analyzed within 1 h after collection according to the WHO guidelines assessing number of sperms per facial field and motility (%). Samples with no visible sperm under the microscope and a Johnsen Score ˂7 (determined by a pathologist (Johnsen 1970)) were cryopreserved only if patients insisted.

A sample’s banking status was categorized as “ongoing storage”, “transferred to other fertility centres”, “electively discarded”, or “discarded because of patient’s death”.

### Assessment of fertility outcome

We assessed maturation, fertilization, pregnancy, miscarriage, and live birth rates following ART with cryopreserved samples. WHO definitions were used to describe perinatal outcomes (gestational age, weight, and height at delivery (World Health Organization 2004).

### Statistical methods

Data analysis was conducted using R, version 3.6.1. All continuous variables are presented as means and standard deviations (SD) or median values and interquartile ranges (IQR). Comparisons with numerical variables were made using the *one-way ANOVA* or *Kruskal–Wallis* test depending on data distribution. *p* values < 0.05 were considered statistically significant.

## Results

### Patient characteristics and description of samples

**Fig. 1:**
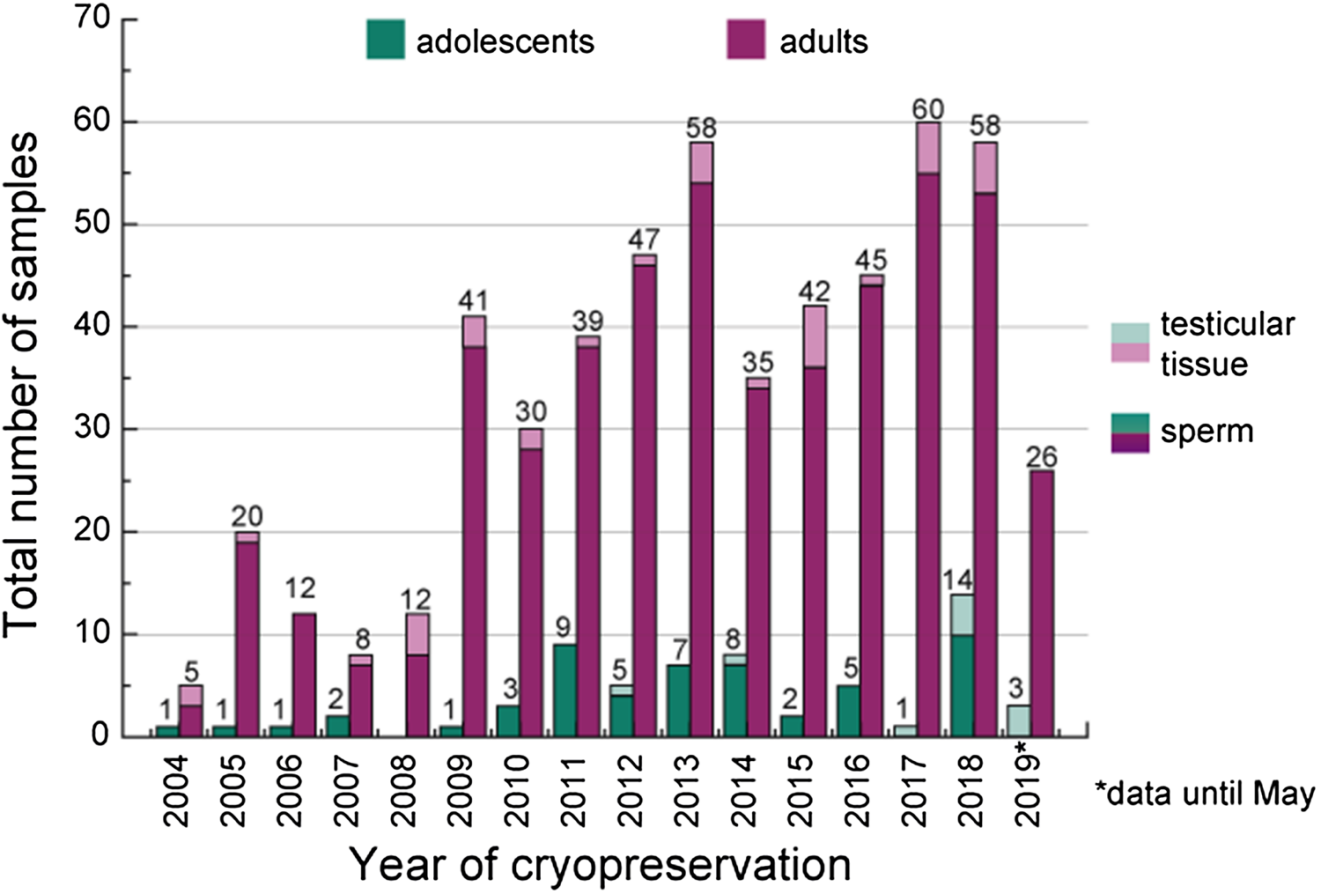
Annual numbers of sperm and testicular tissue samples cryopreserved by male adolescent and adult cancer patients between 03/2004 and 05/2019, shown by year of cryopreservation. Overall, 601 samples were stored, of which 554 were sperm (501 adults and 53 adolescents) and 47 testicular tissue samples (37 adults and 10 adolescents)

Out of 506 cancer patients, 53 (10.5%) were younger than 18 years old at cancer diagnosis (mean age total population 29.4 ± 9.1 years). Patient and sample characteristics are presented in Table 1. In total, patients had cryopreserved 601 samples, of which the majority was collected before the initiation of an oncologic treatment (76.7%, 460/600). While both adolescents and adults had mainly banked semen, the rate of testicular tissue cryopreservation was higher in adolescents compared to adults before (11.8%, 6/51 vs. 6.4%, 26/406) and following the start of cancer treatment (16.7%, 4/24 vs. 7.8%, 13/167). Generally, the number of adults who cryopreserved in our center increased over the years (2004–2019), whereas annual numbers of samples provided by adolescents were constantly low (Fig. 1). Semen analyses revealed normospermia in 52.5% (290/552) of patient’s samples. In testicular tissue, spermatozoa were found in 66.7% (28/42) of samples. According to the examination specifications from Johnson et al. 1980, at least 25 tubules of testicle tissue samples were subjected to histometric analysis in the pathology department to assess daily sperm production and thus the degree of maturity of spermiogenesis (Johnsen Score). The Johnsen Score was determined in 15 samples, out of which 7 (46.7%) had a Johnsen Score of 8 or more. At the time of study conduction, storage had already been ended for almost half of all samples collected (44.8%, 269/601). Of these, more than half had been disposed on the patient’s behalf (59.3%, Table 2). A total of 46 patients had previously requested their samples for ART (9.1%), and most (71.5% (28/39) within the first three years after cryopreservation (Supplement, Fig. 2).

**Table 1:** Characteristics of cancer patients who cryopreserved semen and/or testicular tissue between 03/2004 and 05/2019 at Charité-Universitätsmedizin Berlin

Variables	MD	Cancer diagnosis									
		Hematological malignancy		Brain tumor		Testicular tumor		Non-testicular tumor		Total	
Nº of cancer patients, *n* (%)	–	191	37.7	18	3.6	192	37.9	105	20.8	**506**	–
Mean age at diagnosis [SD]	15	27.76 [9.56]	–	23.18 [6.53]	–	29.87 [6.73]	–	32.30 [11.04]	–	29.36 [9.05]	
< 18, *n* (%)	2	33	17.4	3	16.7	4	2.1	13	12.5	53	10.5
≥ 18, *n* (%)		157	82.6	15	83.3	188	97.9	91	87.5	451	89.5
Mean age at the 1st cryopreservation [SD]	0	27.85 [9.57]	–	22.88 [5.77]	–	29.93 [6.72]	–	32.55 [10.92]	–	29.44 [9.04]	–
Range age at the 1st cryopreservation	0	[9–71]	–	[15–38]	–	[16–48]	–	[14–67]	–	[9–71]	–
Mean nº of collections per patient [SD]	0	1.20 [0.42]	–	1.19 [0.38]	–	1.19 [0.43]	–	1.16 [0.37]	–	1.19 [0.41]	–
Nº of samples cryopreserved	–	230	38.3	21	3.5	228	37.9	122	20.3	**601**	–
Sperm samples, *n* (%)	0	221	96.1	19	90.5	202	88.6	112	91.8	554	92.2
Mean age at the 1st cryopreservation [SD]	0	27.75 [9.30]	–	29.95 [5.47]	–	29.63 [6.25]	–	32.53 [10.74]	–	29.23 [8.79]	–
Normozoospermia (%)	2	121	55	11	57.9	93	46.0	65	58.6	290/552	52.5
Oligo/asthenozoospermia (%)		60	27.3	5	26.3	59	29.2	31	27.9	134/552	24.3
Oligoasthenozoospermia		39	17.7	3	15.8	50	24.8	15	13.5	128/552	23.2
Testicular tissue samples, *n* (%)	0	9	3.9	2	9.5	26	11.4	10	8.2	47	7.8
Mean age at the 1st cryopreservation [SD]	0	20.22 [10.57]	–	19.5 [6.36]	–	32.92 [8.08]	–	33.3 [13.19]	–	30.00 [10.98]	–
Sperm found	5	1/8	12.5	1/1	100	17/24	70.8	9/9	100	28/42	66.7
Mean Johnsen Score (JS) [SD]	32	5.50 [3.07]	–	–	–	5.79 [3.10]	–	6.21 [3.09]	––	6.33 [3.01]	–
Johnsen Score (JS) ≥ 8	32	1/3	33.3	–	–	5/7	71.4	2/5	40	7/15	46.7
Chemotherapy and/or HSCT (yes)—n (%)	29	226	99.1	20	100	137	66.8	103	86.6	486	85.0
Radiotherapy (yes), *n* (%)	22	69	30.8	13	65	17	7.8	49	41.2	148	25.6
Surgery (yes), *n* (%)	2	4	1.8	16	76.2	225	98.7	32	26.2	277	46.1
Brain, *n* (%)	5	0	0	15	93.8	0	0	1	3.6	16	5.9
Pelvis, *n* (%)	5	0	0	0	0	5	2.2	27	96.4	32	11.8
Orchiectomy (at least one testicle) (%)	5	4	100	0	0	220	97.8	0	0	224	82.4
Cryopreservation before treatment (%)	1	183	79.9	6	28.6	176	77.2	95	77.9	460	76.7
Time of cryopreservation until 1st cancer treatment (days) [IQR]	78	3 [1–6.5]	–	44 [1–91]	–	8 [0–29.25]	–	3 [1–7.25]	–	3 [1–14]	–
Cryopreservation after starting treatment (%)	1	46	20.1	15	71.4	52	22.8	27	22.1	140	23.3
Time of cryopreservation since the 1st treatment (days) [IQR]	17	14 [3–163.5]	–	32 [14.5–79]	–	28 [13.75–93.5]	–	21 [3.75–87]	–	22 [5–132]	–

*MD* missing data; *SD* standard deviation; *IQR* interquartile range; *HSCT* hematopoietic stem cell transplantation

**Table 2:** Status of samples cryopreserved between 03/2004 and 05/2019 at time-point of study conduction (01/2020–09/2021)

	Sample’s banking status										
Variables	MD	Ongoing storage (%) *n = *332 samples (55.2%)				Not ongoing storage (%) *n = *269 samples (44.8%)					
		Ongoing full samples		Pick up partially ART		Electively discarded		Fertility center		Patient has deceased	
*N*º of samples cryopreserved n (%)	1	326	54.3	6	1	159	26.5	52	8.7	57	9.5
Mean age at diagnosis [SD]	18	27.26 ± 8.9	–	31 ± 11.9	–	29.81 ± 7.50	–	35.42 ± 7.78	–	32.29 ± 10.58	–
< 18, n (%)	3	48	75	1	1.5	9	14.1	0	0	6	9.4
≥ 18, n (%)		277	51.9	3	0.6	150	28.1	52	9.8	51	9.6
Year at cryopreservation 2004–2008	0	17	28.3	2	3.3	27	45	10	16.7	4	6.7
2009–2013		110	45.6	1	0.4	79	32.8	25	10.4	26	10.8
2014–2019		199	66.6	3	1.0	53	17.7	17	5.7	27	9.0
Mean age at cryopreservation [SD]	0	27.34 ± 8.90	–	31 ± 9.31	–	29.84 ± 7.45	–	35.63 ± 7.86	–	32.81 ± 10.35	–
Range age at cryopreservation	0	[9–53]	–	[17–44]	–	[16–51]	–	[23–57]	–	[16–67]	–
Sperm samples, *n* (%)	0	307	55.5	6	1.1	145	26.2	42	7.6	53	9.6
Testicular tissue samples, *n* (%)	0	19	40.4	0	0	14	29.8	10	21.3	4	8.5
Cryopreservation before treatment (%)	1	251	54.7	6	1.3	126	27.4	33	7.2	43	9.4
Cryopreservation after starting treatment (%)		75	53.5	0	0	33	23.6	19	13.6	13	9.3

*MD* missing data; *SD* standard deviation; *ART* assisted reproductive technology

### ART and perinatal outcome

Among the 46 patients who had previously collected a total of 58 samples for ART, only one (2.2%) was an adolescent at time of cancer diagnosis. Mean age at sample collection was 38.0 ± 6.9 years. Patients with non-testicular cancer (17.1%, 18/105) were most likely to collect their samples, followed by patients with hematological malignancies (7.9%, 15/191), testicular tumors (6.3%, 12/192), and brain tumors (5.6%, 1/18). Former testicular cancer patients requested their samples for ART after a shorter period after cryopreservation (19.1 ± 16.0 months, p = 0.521) than those with a non-testicular tumor (25.9 ± 37.9 months) or a hematological malignancy (28.4 ± 40.5 months). Most patients who had requested their samples for ART had cryopreserved these before treatment (71.7%, 33/46). Only 17.24% of samples collected for ART were testicular tissue (17.24%, 10/58).

We received detailed information on 37 fertility cycles conducted in 21 out of the 46 men who underwent ART following collection of their samples stored in our cryobank. None of these men had cryopreserved testicular tissue. Mean age at first ART cycle in these 21 men was 37.7 ± 5.0 years and 33.0 ± 3.5 years in their partners (Table 3). Only two men had used samples that had been cryopreserved following initiation of oncologic treatment (orchiectomy/tumor resection) and 4 men (19.0%) attempted to use fresh sperm which were collected following oncologic treatment for ART. Among men who had used samples cryopreserved before treatment initiation, the majority (76.9%, 10/13) had eventually received a high-gonadotoxic-risk treatment.

**Table 3:** Utilization of cryopreserved samples for fertility treatment, including embryological outcomes and perinatal characteristics

Fertility information	Parameter	MD	Total	%
Patient characteristics	Total number of patients with fertility cycle information	–	21 patients	**–**
	Male age at the first cycle	3	37.67 [5.02]	**–**
	Female age at the first cycle	7	33.00 [3.48]	**–**
	Mean months duration from cryopreservation to pick up	3	24 [26.96]	**–**
	Range months from cryopreservation to pick up		1–119	**–**
Fertility cycle information	Fresh sperm used (%)	0	4^b^/21	19.0
	Cryo sperm used (%)		17/21	81.0
	ART cycles, *n* (%)	0	37	100
	IUI-H, *n* (%)	2	9	24.3
	IVF, *n* (%)		0	0
	ICSI, *n* (%)		28	75.7
	Number of IVF/ICSI/IUI-H cycles 1	1	11/20	55
	Number of IVF/ICSI/IUI-H cycles 2 to 3		9/20	45
	Oocytes’ maturation rate per cycle	1	219/270	81.1
	Fertilization rate	1	111/158	70.3
	Mean number of ET [SD]	1	1.52 [0.58]	**–**
	Pregnancy rate per cycle	0	19/37	51.4
	Pregnancy rate per cycle with cryo samples	0	18/32	56.3
	Miscarriage rate	2	4/35	11.4
	Total offspring born after ART per cycle	1	17/35	48.6
	Live births rate per patient	1	13/20	65
	Live births rate with cryo samples per patient	0	13/17	76.5
	Cycles with day 2–3 ET	4	9/27	33.3
	Live-birth rate per cycle (%)	0	6/9	66.7
	Cycles with day 4 ET	4	4/27	14.8
	Live-birth rate per cycle (%)	0	1 /4	11.4
	Cycles with day 5–6 ET	4	12/27	44.4
	Live-birth rate per cycle (%)		8/12	66.7
Children and delivery information	Total offspring born after ART	1	17	**–**
	Number of pregnancies with multiple siblings	1	3/18	16.7
	Mean weeks at delivery [SD]	2	37.81 [3.11]	**–**
	Preterm birth (< 37 weeks gestation)^a^		3/13	23.1
	Mean weight at delivery [SD]	3	3032.71 [478.12]	**–**
	Low birth weight (< 2500 g)^a^		3/14	21.4
	Mean height at delivery [SD]^a^	8	50.31 [2.40]	**–**
	Birth mode: natural	2	8/12	66.7
	Vaginal operative		1/12	8.3
	Cesarean		3/12	25.0

*MD* missing data; *SD* standard deviation; *ART* assisted reproductive technology; *ICSI* intracytoplasmic sperm injection; *IVF* in vitro fertilization; *IUI-H* homologous intrauterine insemination; *ET* embryo transfer

^a^World Health Organization definitions were employed (https://www.who.int)

^b^Only one began with fresh sperm and change into cryo sperm in the third ART cycle

Overall, 19 pregnancies in 37 cycles (rate 51.4%) were documented in 16 patients. Only one out of the four men who used fresh sperm achieved a pregnancy by homologous intrauterine insemination (IUI-H). Another patient who initially attempted to use fresh sperm showed severe oligoasthenoteratozoospermia in a first and second cycle but eventually achieved a pregnancy following a third cycle using his previously cryopreserved sperm. Pregnancies resulted in 11 singleton live births, three twin pairs, and four miscarriages (which occurred in two patients). Live-birth rate for the whole population was 65% (13/20) and 76.5% (13/17) for the patients who used cryopreserved samples. Complications were reported for two partners during pregnancy: one had preeclampsia, and the second one threat of premature birth. Further perinatal outcome data are shown in Table 3.

## Discussion

We present our monocentric 15-year experience of sperm and testicular tissue cryopreservation in cancer patients. Our study adds information to knowledge on cryopreservation practices in male cancer patients, also addressing adolescent cancer patients, and on outcomes of ART using these cryopreserved samples.

The number of patients who stored samples in our cryobank has grown noticeably from 2004 to 2019. Awareness of cancer treatment-related infertility has increased, particularly in recent years. Resulting in more pronounced attention to FP in clinical standards, and to the introduction of guidelines for childhood, adolescent, and adult cancer patients (Dittrich et al. 2018; Lambertini et al. 2020). The increase of patients undergoing cryopreservation in our cryobank may be attributed to promoting developments of the department itself and/or cooperating institutes. This includes expanding patient education with the help of advertisement and brochures, websites or lectures, improved supply, and growing cooperation with clinics/private practices in the federal states of *Berlin* and *Brandenburg.* We collected retrospective cryopreservation data until 05/2019. A few months later, the legal basis obliging health insurance coverage of FP was achieved in Germany. Since then, an even stronger annual rise in the number of cryopreserved samples in our department is noticeable (2020: *n = *110, 2021: *n = *128, not shown in results). Reports from other countries in which a public funding program has been implemented similarly show increasing cryopreservation rates (Herrero et al. 2016). Yet, funding of cryopreservation is not implemented across all European countries and not all patients have equal access to FP (European atlas of fertility treatment policies 2021). Networks such as the interdisciplinary paneuropean late effect network, *PanCare,* advocate to improve equal chances in treatment for patients throughout Europe (Mulder et al. 2021). In Germany, Austria, and Switzerland, e.g., the *FertiProtekt* network greatly contributes to improved accessibility of FP for cancer patients (FertiPROTEKT Netzwerk 2006).

Recent studies reveal generally low cryopreservation rates among cancer patients (Bizet et al. 2012; Mulder et al. 2021), e.g., in a current study less than half of adolescent cancer patients cryopreserved samples (Balcerek et al. 2020). Barriers in utilization include financial issues, availability of measures, insufficient time for FP due to urgency of cancer treatment, patient’s chance of survival, insufficient advice, cultural and religious beliefs, a previous fatherhood status, or indecision about wanting to be a parent (Halpern et al. 2020). In our study, most cryopreserved samples (75.6%) were collected by either patients with a testicular cancer or a hematological malignancy. These results are similar to previous studies with numbers of utilization ranging from 19 to 41.7% for testicular cancer and 35.4 to 71.7% for hematological malignancies (Bizet et al. 2012; Depalo et al. 2016; Reschini et al. 2021). Among all samples cryopreserved in our department, only 10% were collected from under-aged cancer patients. However, future parenthood is already a topic of relevance in adolescents (Picton et al. 2015). FP poses a particular challenge in children and adolescents with cancer (Picton et al. 2015). While adolescents are being offered long-established sperm and/or testicular tissue cryopreservation in our cryobank, FP in prepubertal patients is only available in experimental settings (Kabiri et al. 2022). Immature testicular tissue cryopreservation in prepubertal boys is a promising technique (Kabiri et al. 2022). We offer this procedure in collaboration with the *Androprotect Study* (*Universitätsklinik Münster, Germany*). Despite advances of reproductive medicine, further knowledge on the specific risks of cancer treatment is still required to improve individual counseling and FP strategies for cancer patients.

Due to impaired fertility following cancer treatment, a rising number of cancer survivors use ART to fulfill their desire for a child of their own (Verona et al. 2021). In Germany, almost twice as many survivors reported having conceived following ART than rates published for the general population (2.6 vs. 4.6%) (Sommerhäuser et al. 2021). In our study, only 9.1% of cryopreserved samples were eventually used for ART, which is comparable with the aggregated usage rate of 8% published by Ferrari and colleagues who reviewed 30 studies (Ferrari et al. 2016). Some of the reasons for survivors for not having used their cryopreserved samples included having achieved conceptions naturally, no desire to have a child (yet) or follow-up period after cryopreservation being too short for especially adolescents. In our study, most samples that were requested for ART had been cryopreserved before a high-gonadotoxic-risk cancer treatment. The majority of patients had used their samples within the first 3 years following cryopreservation, with utilization rates further decreasing from 4 to 12 years since cryopreservation. Similar trends were reported previously (Depalo et al. 2016), with none of the cryopreserved samples having been used after a follow-up period of 15 years (Kelleher et al. 2001). Patients in our study collected their samples at a mean age of 38.00 ± 6.87 years, which may be related to a generally higher age of first fatherhood in European countries (average 34.6 years) (Federal Statistical Office of Germany 2020) and to the observation that cancer patients tend to reach milestones later in life compared to peers (Langeveld et al. 2003). Only one adolescent cancer survivor had collected his samples for ART. However, it needs to be noted that mean age at diagnosis of adolescent cancer patients in our cohort was 16 ± 1.5 years, and at study time 21 ± 4.2 years, suggesting that follow-up time was too short to examine utilization rate for these patients. Although freezing and thawing procedures potentially decrease sperm motility and total motile sperm count (Kelleher et al. 2001), most patients in our cohort successfully achieved pregnancies using their cryopreserved sperm (56.3%), of which 76.5% resulted in live births. These results are similar to other studies (Fu et al. 2019), which reported a pregnancy rate of 51.5% (17/33) and a live birth rate of 71.4% (10/14) following ART with cryopreserved samples. Half of our patients and their partners only required one ART cycle to achieve a pregnancy, and none required more than three cycles, which is reassuring. In our cohort, in line with results of other studies (Depalo et al. 2016), the majority of patients underwent ICSI (75.7%), which is more efficient in case of severe male factor infertility compared to IVF (Haddad et al. 2021), and reduces the risk of failed fertilization (Depalo et al. 2016). In our population, only one pregnancy was reported after the use of fresh sperm. Adverse perinatal outcomes occurred in a fifth to a quarter of pregnancies, such as prematurity (23.1%), low birth weight (21.4%), and/or cesarean section (25.0%), which can be associated with the higher prevalence of multiple sibling births following ART compared to following natural conception (Sommerhäuser et al. 2021). In our cohort, 21.4% of births following ART were multiple sibling births. Nowadays, fewer embryos are used per transfer than at the beginning of the observation period, and consequently, the multiple-birth rate has been reduced (Verona et al. 2021). ART in cancer patients has not been associated with increased risk of congenital abnormalities or adverse health outcomes (Picton et al. 2015).

### Study limitations

Limitations regarding our study design need to be taken into account. Due to the retrospective setting, we were only able to collect information on those patients who cryopreserved their samples in our cryobank. However, no information was available on patients who ultimately did not store samples; similarly, we cannot provide reasons for why these samples were not stored.

Moreover, it should be noted that the clinical practice of FP recommendations and oncological treatment strategies have changed over the years of the retrospective study period. Unfortunately, we saw, that despite these changes, numbers of cryopreservation in adolescents remained low over the period of 15 years. Specifically adolescents would, however, benefit from FP and enhance their chances of a future parenthood. In accordance to guideline recommendations, the majority of samples were cryopreserved before start of the treatment. Therefore only little information is available on samples collected after treatment. Information on outcome using these samples is relevant for counseling patients who are unable to cryopreserve before initiation of a gonadotoxic treatment. No follow-up on natural conceptions was documented for patients. As we do not provide in-house ART, we could only address those patients to provide information on ART cycles who had previously requested their samples from our department. ART was conducted in external fertility centers chosen by the patients, resulting in variances of ART procedures between centers. Future studies should examine the success of ART using testicular tissue samples, which was not possible in the current study as none of the patients had requested their testicular tissue for ART.

## Conclusions

We present our monocentric experience of FP in male cancer patients, including adolescents. As recommended in guidelines, the majority of patients had cryopreserved sperm samples and/or testicular tissue samples prior to cancer treatment. Cryopreservation rates among adolescents were low with little increase over time. Overall, only 10% of samples cryopreserved were used for ART. Existing fears of using cryopreserved samples for ART, and success rates, should be discussed in detail with patients. Our results of outcomes following ART with cryopreserved samples are reassuring regarding the efficiency and perinatal outcomes. However, outcomes need to be confirmed in a larger cohort. With the new advantage of public health insurance coverage of FP in Germany, the number of those turning to cryopreservation in the context of potential gonadotoxic treatment has already risen, emphasizing the necessity of further knowledge for individual counseling.

## Supplementary Information

Below is the link to the electronic supplementary material.

Supplementary file1 (DOCX 59 kb)

## Data Availability

The data underlying this article will be shared on reasonable request to the corresponding author.
