# Supplementary material for: Sperm and testicular tissue cryopreservation and assisted reproductive technology outcomes in male cancer patients: a 15-year experience
Source: J Cancer Res Clin Oncol. 2022 Nov 23;149(8):5321–30. doi: 10.1007/s00432-022-04488-y (PMC10349735; doi:10.1007/s00432-022-04488-y)

**Supplement Figure 1.** CONSORT diagram on cohort enrollment and exclusion criteria

**Sperm and testicular tissue cryopreservation
and assisted reproductive technology outcome in male cancer patients: a 15-year experience**

**919 males** cryopreserved
**1073 samples** (sperm and/or testicular tissue)
March 2004 - May 2019

**603 cancer patients** and **733 samples**

**506 cancer patients**, with information on cancer therapy, cryopreserved **601 samples**

**46 cancer patients used their**

**samples for fertility treatment**

(9%)

26 patients gave consent for collecting fertility treatment data from fertility centers (56%)

4 patients without information from fertility centers

21 patients with information on fertility cycles

Contacted patients (by mail, email or phone):

n=16 no valid contact information available/no answer/died

n=4 declined participation

Excluded for analyses:

n=332 patients ongoing storage

n=159 stopped the contract

n=57 patients who had deceased

Excluded for analyses:

n=97 patients and 132 samples with missing information on cancer treatment

Excluded for analyses:

n=185 samples with no information on diagnosis

n=155 samples of patients with no cancer diseases

**Supplement Figure 2.** Years from cryopreservation to pick-up shown in reference to age at cryopreservation in patients (n=39) who requested their cryopreserved samples for fertility treatment.


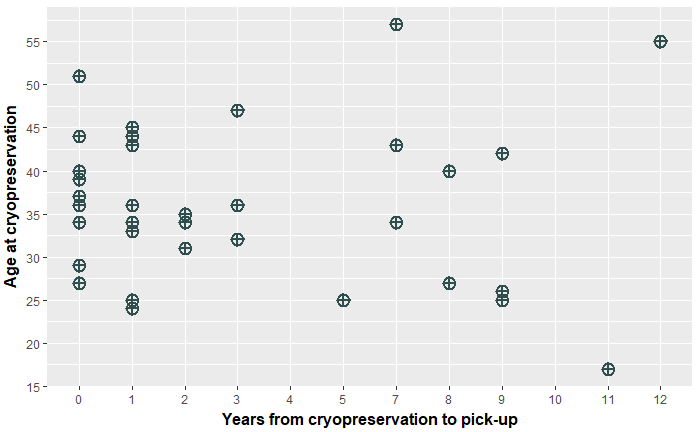

Supplement: Supplementary file 1 — Supplementary file1 (DOCX 59 kb) [file 432_2022_4488_MOESM1_ESM.docx]
